# Topical administration of *Juglans regia* L. leaf extract accelerates diabetic wound healing

**DOI:** 10.1186/s12906-022-03735-6

**Published:** 2022-10-03

**Authors:** Davood Nasiry, Ali Reza Khalatbary, Alireza Ghaemi, Mohammad Ali Ebrahimzadeh, Mohammad Hossein Hosseinzadeh

**Affiliations:** 1 Amol Faculty of Paramedicine, Mazandaran University of Medical Sciences, Sari, Iran; 2 Cellular and Molecular Research Center, Mazandaran University of Medical Sciences, Sari, Iran; 3 Department of Nutrition, Health Sciences Research Center, Faculty of Public Health, Addiction Institute, Mazandaran University of Medical Sciences, Sari, Iran; 4 Pharmaceutical Sciences Research Center, Faculty of Pharmacy, Mazandaran University of Medical Sciences, Sari, Iran; 5 Faculty of Pharmacy, Mazandaran University of Medical Sciences, Sari, Iran

**Keywords:** Diabetic wound, Wound healing, *Juglans regia* leaf extract, Collagen biosynthesis

## Abstract

**Background:**

Diabetic wounds are one of the most important issues in diabetic patients. It seems that *Juglans regia* L. leaf with antioxidant and anti-inflammatory potentials can be profitable for healing of diabetic wounds. The aim of present study was to investigate the topical administration of *Juglans regia* L. leaf extract in diabetic wound healing.

**Methods:**

Seventy-five diabetic male rats were randomly divided into 5 groups (*n* = 15), including: untreated (Control) group, Eucerin group, 2% *Juglans regia* L*.* ointment (JRL 2%) group, 5% *Juglans regia* L*.* ointment (JRL 5%) group, and Phenytoin group as a reference drug. Sampling was performed at days 7, 14, and 21 after surgery. Evaluation tests included stereology, immunohistochemistry, molecular, and biomechanical.

**Results:**

Our results showed that the wound closure rate, volumes of newly formed of epidermis and dermis, density of fibroblasts and blood vessels, collagen deposition, density of proliferation cells, expression levels of TGF-β and VEGF genes, and biomechanical characteristics were significantly higher in extract groups compared to control and eucerin groups, however, these changes were considerable in the JRL 5% group (*P* < *0.05*). This is while that the density of neutrophils and expression levels of TNF-α and IL-1β genes in the extract groups, especially in the JRL 5% group, were significantly reduced compared to control and eucerin groups (*P* < *0.05*).

**Conclusion:**

Topical administration of *Juglans regia* L. leaf extract, especially in 5% concentration, considerably accelerates diabetic wound healing.

**Supplementary Information:**

The online version contains supplementary material available at 10.1186/s12906-022-03735-6.

## Background

Diabetic wound management and treatment has always been one of the most important challenges in diabetic patients [1]. About 34 percent of people with diabetes suffer from diabetic foot ulcers, and about 84 percent of cases lead to foot amputation [2]. Wound healing process is a spontaneous event that mediated through several sequential mechanisms [3]. Wound healing process starts with hemostasis, during which platelet plaque is formed and prevents bleeding from the injury site. The next stage is inflammation which begins with the influx of neutrophils to the wound site in order to remove debris and prevent infection. This event is enhanced by the release of histamine from mast cells. At this stage, monocytes enter the wound site with a delay compared to neutrophils and differentiate into macrophages to phagocytize the remaining cell debris. The next stage is called the proliferative phase, during which keratinocytes begin to migrate to close the wound site, new blood vessels are created in the site, and fibroblasts replace the initial fibrin tissue with granulation tissue. Macrophages and T lymphocyte cells play a vital role in promoting this stage. At the end, the extracellular matrix is regenerated by fibroblasts and the wound is closed by the contraction of myofibroblasts [4, 5]. However, wound healing process is dysregulated in diabetic ulcers due to various reasons. The first main and important reason is the prolonged inflammation, which is due to the excessive production of inflammatory cytokines such as tumor necrosis factor (TNF)-α and interleukin (IL)-1β from the immune cells present in the site, and this causes a serious disturbance in the function of the another local cells [6]. The next reason is the weakness of local cells such as fibroblasts and keratinocytes due to oxidative stress caused by systemic hyperglycemia in the production of some cytokines effective in repair and regeneration such as transforming growth factor (TGF)-β and insulin-like growth factor (IGF)-1 [7, 8]. Also, due to the weakness of fibroblast cells, the production of collagen fibers as the most important compound in the extracellular matrix (ECM) faces a serious problem and the closing time is delayed [9]. Moreover, due to the inappropriate quality of the produced collagens, even if the diabetic wound heals, it does not have ideal biomechanical properties and after some time, the wound will form again [10]. The next and important reason for the stability of diabetic wounds is the hypoxia of the wound site caused by the disturbance in the angiogenesis process and diabetic vasculopathy [11]. The main and important factor in the occurrence of angiogenesis is vascular endothelial growth factor (VEGF), which is produced by the local cells of the wound [6]. In diabetic wounds, due to the reasons mentioned earlier, the production of this cytokine faces a serious problem [12, 13]. It seems that if it is possible to use a combination that has properties such as availability, anti-inflammatory, antioxidant and antimicrobial, it can be helped to a great extent in the healing of diabetic wounds [14, 15].

In the meantime, herbal medicines have been of interest to the public for a long time, and in recent decades, they have attracted the special attention of researchers [16, 17]. The use of medicinal plants against chemical drugs, in addition to fewer side effects, is economical and safer and more accessible [18]. One of these plants, which is highly recommended in Iranian traditional medicine, is walnut (*Juglans regia* L.) [19–21]. The most important parts of this plant are its leaves, whose main phenolic compounds include flavonoids, phenolic acids and naphthoquinones [22, 23]. Studies have shown that the consumption of *Juglans regia* L. leaf extract causes a significant decrease in blood sugar in diabetic patients [24, 25]. Also, animal studies have shown positive and significant effects of *Juglans regia* L. leaf extract in the prevention and treatment of diabetic complications such as neuropathy [26], nephropathy [27] and retinopathy [28]. In addition, our laboratory previously reported that the use of *Juglans regia* L. leaf extract can attenuates the diabetic complications in the male reproductive structure [29]. However, among the most important main mechanisms for this plant in all studies, significant anti-inflammatory and antioxidant effects along with blood sugar reduction have been listed.

Currently, based on traditional medicine documentation and recently scientific evidence, we investigated the beneficial effects of topical administration of *Juglans regia* L. leaf extract and the underlying molecular mechanisms in diabetic wound as a common serious complication of diabetes.

## Methods

### Plant material and preparation of plant extract

*Juglans regia* L*.* leaves were obtained from the Sari suburb, Iran in august 2021, from 25–26 year old trees. The leaves were dried at room ambient (away from sunlight) and then powdered before extraction. Dried powder (75 g) was extracted using methanol (200 mL) by maceration for one day at room temperature. After filtration through filter paper, the residue was extracted twice more. Extracts were filtered, combined and concentrated under reduced pressure at 34–36 °C using a Heidolph rotary evaporator [26–28]. A 5% and 2% ointment was prepared in Eucerin.

### Animals and experimental design

A total of seventy-five male Wistar rats (200–250 g and 8 weeks old) were obtained from Pasteur Institute in Mazandaran, Iran. In order to induce type 1 diabetes, all animals received a single intraperitoneal (ip) dose of streptozotocin (STZ; 55 mg/kg). Three days later, the diabetes was validated as fasting blood sugar (FBS) > 250 mg/dL, and animals were placed in individual metabolic cages under normal laboratory conditions. Wound creation and treatments were performed after a 30-day period of diabetes. Studies have shown that in order for the symptoms of diabetes in rats to occur, it takes between 21 and 30 days from the onset of diabetes [10, 27, 28]. At the end of day 30, the animals were randomly allocated into five groups (n = 15), including: untreated (Control) group, vehicle treated (Eucerin) group which received topically with 1 g of ointment base, 2% extract ointment (JRL 2%) group which received topically with 1 g of 2% *Juglans regia* L*.* ointment, 5% extract ointment (JRL 5%) group which received topically with 1 g of 5% *Juglans regia* L*.* ointment, and Phenytoin treated group which received topically 1 g of phenytoin as a reference drug. Administration was done daily and at the same times in all groups for 21 days. Sampling of the studied groups was performed on days 7, 14 and 21 days after surgery [10, 30].

### Wound model

The rats were anesthetized using ip injections of ketamine and xylazine (50 and 5 mg/kg, respectively). Then, the rats were placed on a surgical table in a prone position and their back thoracic hairs were shaved. A 15 mm in diameter was cut off from an upper thoracic region on the back of the rats. Wound size was the same in all rats and was performed by an experienced person using a scalpel No. 15 blade. Also, the depth of wounds was full-thickness which included both dermis and hypodermis.

The progressive changes in the wound closure were photographed on days 0, 7, 14, and 21 after surgery, using a digital camera (FinePix S20, Japan). Then, the photos were analyzed using MacBiophotonics in ImageJ software (National Institutes of Health) and wound closure rate was calculated and compared between the groups on each time point [13], using the following formula:

$$\mathrm{Wound}\;\mathrm{closure}\;(\%)=\frac{A_0-A_n}{A_0}\times100$$

where, A_n_: wound area on day n, and A_0_: wound area on day 0 [31].

### Histological and stereological assessments

The animals were deeply anesthetized and sacrificed at the three target time points of the experiment and full-thickness of the wound tissues and normal adjacent skin were harvested. The samples were immediately placed in 10% formalin fixative. After tissue processing, the samples were molded in paraffin and then serial sections were prepared using microtome. The thickness of the sections was in two sizes, 5 μm (to assess the volumes of the newly formed epidermis and dermis and the density of collagen) and 20 μm (to evaluate the density of fibroblasts, neutrophils and blood vessels). The number of selected sections from each sample were 10 at equal intervals [32]. Next, the sections were stained using hematoxylin and eosin (H&E) and Mallory’s trichrome (MT). The distribution of collagen, in the MT-stained sections, was quantified using MacBiophotonics in ImageJ software (National Institutes of Health) and digital densitometry recognition. For this purpose, a total of 25 randomly picked micrographs from 5 sections were captured for each sample using a 40 × magnification objective lens. The collagen percentage was calculated by dividing the total blue-colored area by the total areas of the micrographs [13].

#### The volumes of the newly formed epidermis and dermis

To evaluate the volumes of the newly formed epidermis and dermis, Cavalieri method was used. For this purpose, after selecting 10 photos (one photo from each section) of each rat, a grid of points was projected on the photos (Supplementary Fig. 1). Next, all the points that were superimposed on the newly formed tissues were counted. The total volumes of the newly formed epidermis or dermis were evaluated using the following formula:${V}_{total}=\sum P\times \frac{a}{p}\times t$: ΣP: the total number of points counted from 10 photos; a/p (mm^2^): the area related with each square formed between 4 points; and t (mm): the intervals between the selected sections perceivably [10].

#### Density of cells

To estimate density (N_v_) of the fibroblasts and neutrophils in the wound site, the optical dissector method was used (Supplementary Fig. 1). For this purpose, after counting cells in tissue Sects. (10 per rat), the following formula was used: ${N}_{v}=\frac{\sum Q}{\sum P\times h\times \frac{a}{f}}\times \frac{t}{BA}$, where ΣQ: the total number of nuclei; Σp: the total number of the counted frames; h (µm): height of the dissector; a/f (mm^2^): frame area; t (µm): real sectional thickness; and BA (µm): block advance of the microtome (set at 20 μm).

#### Density of blood vessels

The following formula was used to measure the density of blood vessels in the newly formed dermis: ${L}_{v}=\frac{2\sum Q}{\sum P\times \frac{a}{f}}$,: ΣQ: the total number of the counted blood vessels; ΣP: the total number of the counted frames; and a/f (mm^2^): counting frame area (Supplementary Fig. 1).

### Immunohistochemistry

To determine the proliferating cells in newly formed dermis, immunostaining against ki67 antibody was performed [10]. Briefly, ten selected sections with equal distances from each rat were exposed to goat serum for 30 min. Then, anti-ki67 rabbit polyclonal antibody was added to the samples (1: 100 in PBS, Abcam ab833) and incubated overnight at refrigerator temperature. The next day, the sections were exposed to the secondary antibody (goat anti-rabbit IgG-HRP, Abcam) for one hour. Finally, diaminobenzidine tetrahydrochloride (DAB) was added to detect positive reactions. The sections were mounted and evaluated using a light microscope. For quantitative analysis, 5 photos of each sample were collected from all rats in each study group and evaluated by densitometry using MacBiophotonics in ImageJ software (National Institutes of Health). Data are represented as a percentage of total tissue area [33].

### qRT-PCR

In the present study, the expression levels of four genes were examined, including TGF-β (effective in proliferation and regeneration), VEGF (effective in angiogenesis), TNF-α and IL-1β (inflammatory genes). For this purpose, the harvested tissue samples on day 7 were homogenized using Lyser device and the total RNA was extracted using Yekta (Yekta-tajhiz, Tehran). The quality of the extracted RNA was confirmed using a Nano-spectrophotometer and 1% gel agarose electrophoresis. The cDNA was reverse-transcribed from 1 µg of the total RNA in a 20 μl reaction mixture based on a protocol derived from Yekta-cDNA synthesis kit (Yekta-tajhiz, Tehran). The ratio of the absorbance at 260 and 280 nm (A260/280) for all samples were about 1.808–2.014, and A260/230 ratios were about 0.3–0.7. Then, qRT-PCR reactions were performed for three biological replicates on the real-time PCR system (Applied Biosystems StepOne instrument) using SYBR Green Master Mix and sets of primers (Table 1). The final analyses were performed using the comparative CT method (2^−ΔΔct^) [13, 34].

**Table 1:** Sequences of primers used to analyze molecular levels

Gene	Sequence (5 ' > 3 ')
TGF-β	F: GGCTGAACCCGAGAGACGGA R: CCATGCGAAGCAGGAAGGGT
VEGF	F: ATCGAAAGTGGTCCCAG R: CAATACTGCGCCGAGTAG
TNF-α	F: AGCGATCTTCTCATTCCTGCTC R: GTTTGCTACGACGCGAGCTAC
IL-1β	F: GACAAGCAAGCACAAAATCCC R: TGGGCTATTGTTTGTGATCCAC
β-Actin	F: CCCATCGATGAGGGTTACGC R: TTTAATGTACCGCAAGATTTC

### Wound strength assessment

In order to evaluate the consistency of the restored tissue, at the end of the study (day 21), a standard rectangular sample measuring 5 × 50 mm was removed from the wound area. The strip samples were placed in a biomechanical testing system (Santam Co., Iran). Deformation rate was maintained at 10 mm/min and the maximum force (N) and energy absorption (J) were calculated.

### Statistical analysis

All quantitative data were analyzed using One-way ANOVA followed by Tukey’s post-hoc tests by SPSS software (version 19, Chicago, IL). The data were expressed as the mean ± SD and the *P* < 0.05 was considered significant.

## Results

### Wound closure rate

**Fig. 1:**
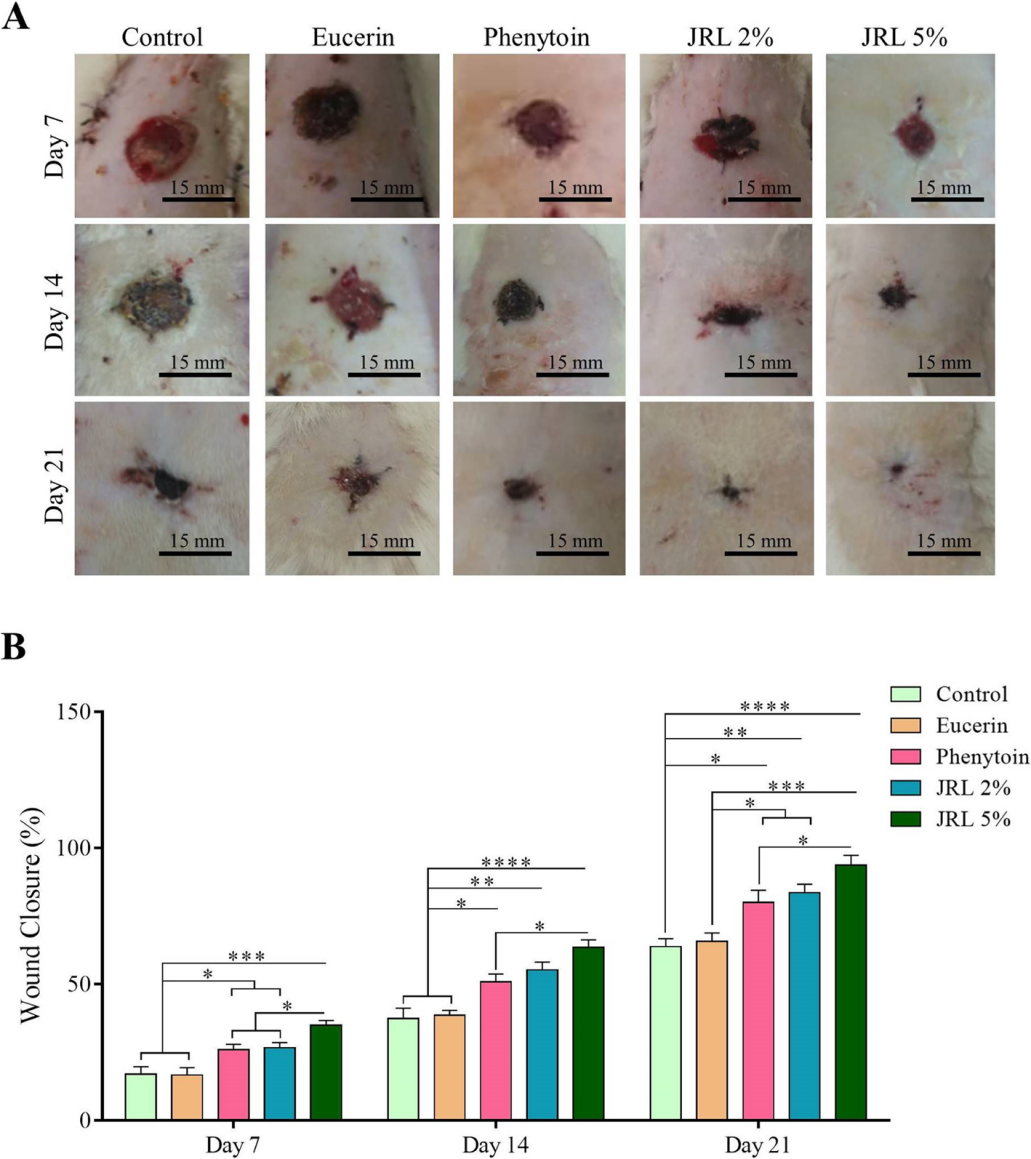
Effects of topical administration of *Juglans regia* L. leaf extract on observational changes and wound closure. **A** The photographs show the progressive healing of the wounds among the study group. **B** Percentage of wound closure on days 7, 14 and 21. Data are represented as Mean ± SD. *P < *0.05*; **P < *0.01*, ***P < *0.001*, ****P < *0.0001*

The photographs of progressive healing of the wounds and their quantitative measurement among the experimental groups on days 7, 14 and 21 are shown in Fig. 1A-B. Evaluating wound closure rate on day 7 showed that phenytoin, JRL 2%, and JRL 5% groups had a significantly higher healing rate in comparison to control and eucerin groups (both, *P* < *0.05*, *P* < *0.05*, and *P* < *0.001*, respectively). Also, among the treated groups on day 7, the wound closure rate in JRL 5% group was significantly higher compared to phenytoin and JRL 2%, groups (both, *P* < *0.05*). Wound closure rate on day 14 was significantly higher in phenytoin, JRL 2%, and JRL 5% groups compared to control and eucerin groups (both, *P* < *0.05*, *P* < *0.05*, and *P* < *0.001*, respectively) (*P* < *0.05*, *P* < *0.01*, and *P* < *0.0001*, respectively). Also, on day 14, the healing rate was higher in the JRL 5% group compared to the phenytoin group (*P* < *0.05*). Finally, evaluation of wound closure on day 21 showed that phenytoin, JRL 2%, and JRL 5% groups had a considerable healing rate compared to control (*P* < *0.05*, *P* < *0.01*, and *P* < *0.0001*, respectively) and eucerin (both, *P* < *0.05*, *P* < *0.05*, and *P* < *0.001*, respectively) groups. Furthermore, the rate of wound closure on day 21 in JRL 5% group was significantly higher than phenytoin group (*P* < *0.05*) (Fig. 1B).

### Stereological parameters

**Fig. 2:**
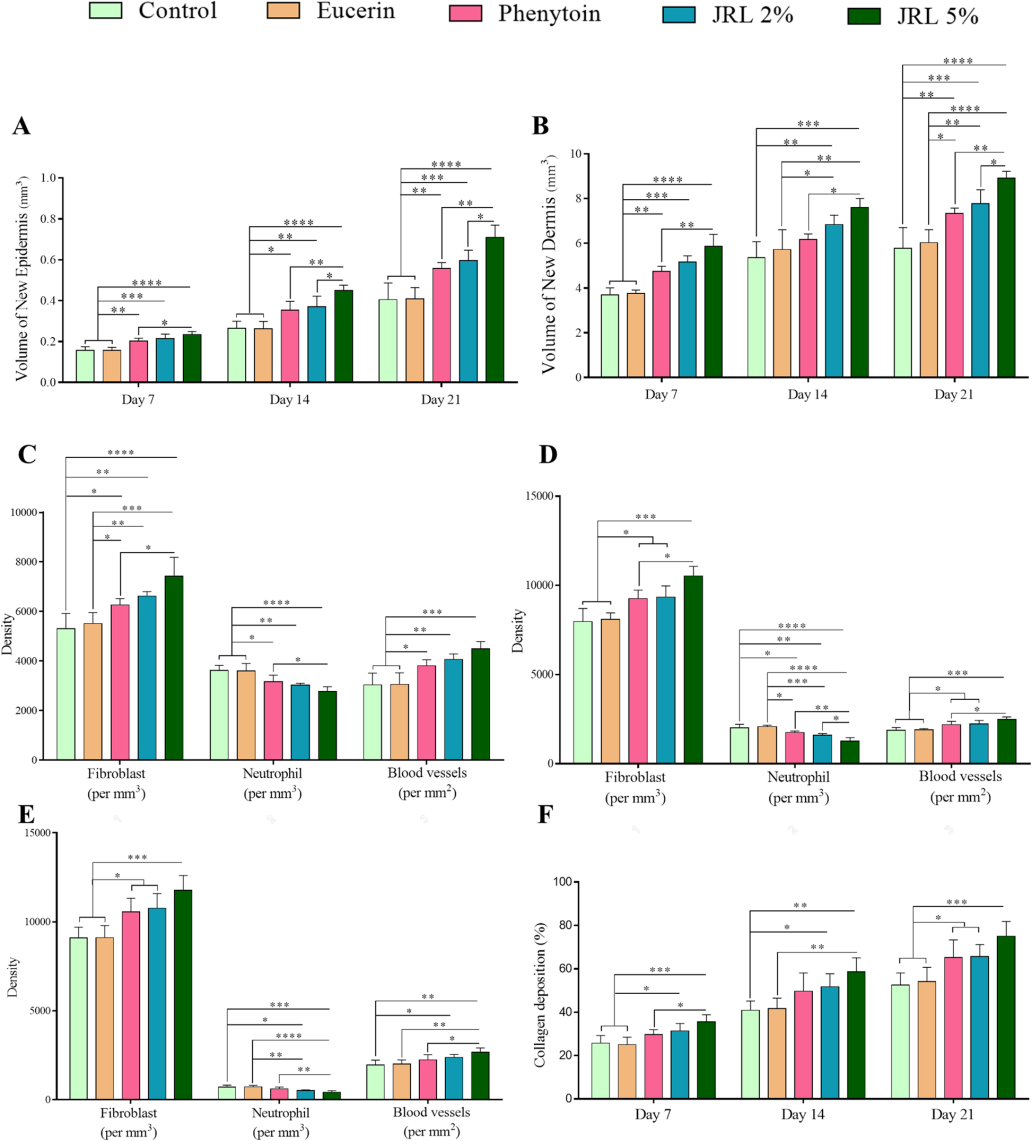
Effects of topical administration of *Juglans regia* L. leaf extract on stereological parameters and collagen density. **A**, **B** Volumes of newly formed epidermis and dermis in the healing wounds on days 7, 14, and 21. Density of fibroblasts, neutrophils and blood vessels in new dermis on days 7 (**C**), 14 (**D**), and 21 (**E**). **F** Collagen density percentage in new dermis on days 7, 14 and 21. Data are represented as Mean ± SD. *P < *0.05*; **P < *0.01*, ***P < *0.001*, ****P < *0.0001*

The results of stereological evaluations are shown in Fig. 2.

#### Volume of newly formed epidermis and dermis

In assessing new epidermis volume on day 7, our finding showed significantly greater volume in phenytoin, JRL 2%, and JRL 5% groups compared to control and eucerin groups (both, *P* < *0.01*, *P* < *0.001*, and *P* < *0.0001*, respectively). Furthermore, comparing the finding between treated groups showed that JRL 5% group had considerably more new epidermis volume than phenytoin groups (*P* < *0.05*). Evaluation of new epidermis volume on day 14 showed that phenytoin, JRL 2%, and JRL 5% groups had significantly more volume than control and eucerin groups (both, *P* < *0.05*, *P* < *0.01*, and *P* < *0.0001*, respectively). Also, among the treated groups, JRL 5% group was significantly higher than phenytoin and JRL 2% groups (*P* < *0.01* and *P* < *0.05*, respectively). Finally, the volume of new epidermis on day 21 was higher in phenytoin, JRL 2%, and JRL 5% groups compared to control and eucerin groups (both, *P* < *0.01*, *P* < *0.001*, and *P* < *0.0001*, respectively). Meanwhile, JRL 5% group had significantly more new epidermis volume than phenytoin and JRL 2% groups (*P* < *0.01* and *P* < *0.05*, respectively) (Fig. 2A).

In measuring of the new dermis volume on day 7, we found that phenytoin, JRL 2%, and JRL 5% groups had significantly higher volume compared to control and eucerin groups (both, *P* < *0.01*, *P* < *0.001*, and *P* < *0.0001*, respectively). In addition, volume of new dermis in JRL 5% group was considerably higher in comparison to phenytoin group, on day 7 (*P* < *0.01*).

Comparing the new dermis volume between experimental groups on days 14 indicated that JRL 2% group in comparison to control and eucerin groups (*P* < *0.05* and *P* < *0.01*, respectively), and JRL 5% compared to control, eucerin, and phenytoin groups (*P* < *0.01*, *P* < *0.0001*, and *P* < *0.05*, respectively) had significantly more volume. Finally, measuring the volume of newly formed dermis on day 21 showed that phenytoin, JRL 2%, and JRL 5% groups were significantly higher compared to the control (*P* < *0.01*, *P* < *0.001*, and *P* < *0.0001*, respectively) and phenytoin (*P* < *0.05*, *P* < *0.01*, and *P* < *0.0001*, respectively) groups. Also, JRL 5% group had significantly more new dermis volume than phenytoin and JRL 2% groups, on day 21(*P* < *0.01* and *P* < *0.05*, respectively) (Fig. 2B).

#### Density of cells and blood vessels

Density of fibroblasts was considerably higher in phenytoin, JRL 2%, and JRL 5% groups compared to control (*P* < *0.05*, *P* < *0.01*, and *P* < *0.0001*, respectively) and eucerin (*P* < *0.05*, *P* < *0.01*, and *P* < *0.001*, respectively) groups, on day 7. Also, evaluation of fibroblast density in two other time periods among the study groups showed that phenytoin, JRL 2%, and JRL 5% groups had significantly higher density in comparison to control and eucerin groups (both, *P* < *0.05*, *P* < *0.05*, and *P* < *0.001*, respectively). Furthermore, JRL 5% group has significantly more fibroblasts density compared to phenytoin group, on days 7 and 14 (both, *P* < *0.05*) (Fig. 2C-E).

Considering neutrophils, we found that the cell density decreased considerably in phenytoin, JRL 2%, and JRL 5% groups compared to control and eucerin groups on days 7 (both, *P* < *0.05*, *P* < *0.05*, and *P* < *0.001*, respectively). On day 14, our results showed significantly lower density of neutrophils in phenytoin, JRL 2%, and JRL 5% groups compared to control (*P* < *0.05*, *P* < *0.01*, and *P* < *0.0001*, respectively) and eucerin (*P* < *0.05*, *P* < *0.001*, and *P* < *0.0001*, respectively) groups. Finally, the evaluation of the density of these cells on day 21 showed that only two JRL 2%, and JRL 5% groups had significantly low density compared to the control (*P* < *0.05* and *P* < *0.001*, respectively) and phenytoin (*P* < *0.01* and *P* < *0.0001*, respectively) groups. Meanwhile, JRL 5% group has significantly low neutrophils density compared to phenytoin group on days 7, 14, and 21 (*P* < *0.05*, *P* < *0.01*, and *P* < *0.01*, respectively) and compared to JRL 2% group on days 14 (*P* < *0.05*) (Fig. 2C-E).

In addition, comparison of blood vessels density showed that there was significantly more vasculature in phenytoin, JRL 2%, and JRL 5% groups in comparison to control and eucerin groups on days 7 (both, *P* < *0.05*, *P* < *0.01*, and *P* < *0.001*, respectively) and 14 (both, *P* < *0.05*, *P* < *0.01*, and *P* < *0.001*, respectively). Moreover, on day 14, blood vessels density in JRL 5% group was significantly more in comparison to phenytoin group (*P* < *0.05*). The evaluation of blood vessels density on day 21 indicated that the JRL 5% group had more density compared to the control, eucerin and phenytoin groups (*P* < *0.01*, *P* < *0.01*, and *P* < *0.05*, respectively). Also, on the same day, the density of blood vessels in the JRL 2% group was higher compared to the control group (*P* < *0.05*).

### Collagen density in the new dermis

To evaluate the collagen density in the newly formed dermis, MT staining was performed. The results of quantification are shown in Fig. 2F. The results showed that deposition of collagen considerably increased in JRL 2% and JRL 5% groups compared to control and eucerin groups, on day 7 (both, *P* < *0.05* and *P* < *0.001*, respectively). Moreover, on day 7, collagen density in JRL 5% group was significantly more in comparison to phenytoin group (*P* < *0.05*).

On day 14, JRL 5% group compared to control and eucerin groups (both, *P* < *0.01*) and JRL 2% group compared to eucerin group (*P* < *0.05*) had significantly more collagen density.

Finally, evaluation of collagen density on day 21 showed that phenytoin, JRL 2%, and JRL 5% groups had higher collagen density than control and eucerin groups (both, *P* < *0.05*, *P* < *0.05*, and *P* < *0.001*, respectively).

### Proliferation of cells in the new dermis

**Fig. 3:**
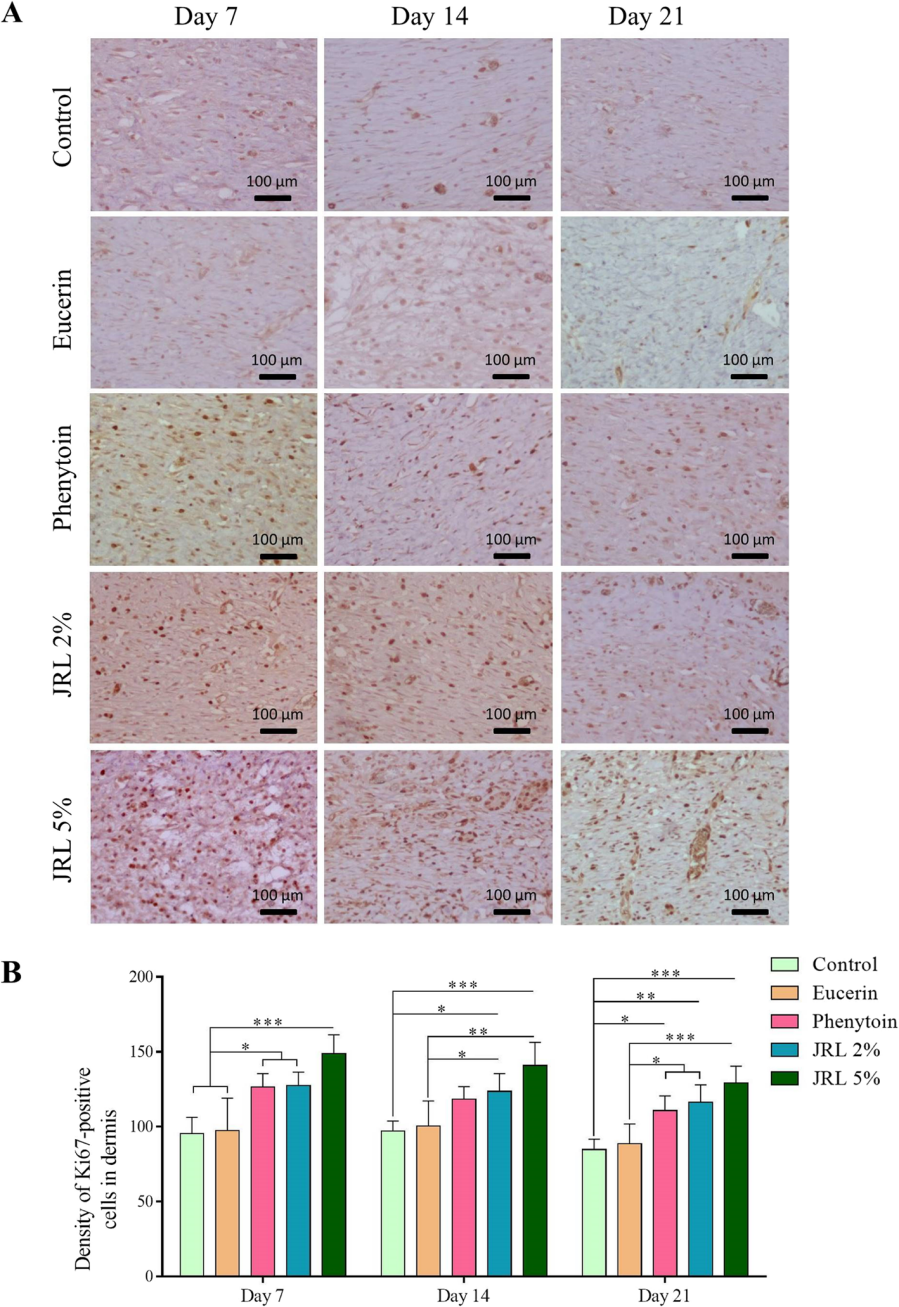
Effects of topical administration of *Juglans regia* L. leaf extract on cell proliferation in new dermis. **A** Immunohistochemical photomicrographs against Ki67 protein (with dark brown nuclei) in new dermis on days 7, 14, and 21. **B** Density of positive stained cells in new dermis in the three time periods studied. Data are represented as Mean ± SD. *P < *0.05*; **P < *0.01*, ***P < *0.001*

In order to evaluate the effects of *Juglans regia* L. leaf extract in the proliferation of cells in the new dermis, immunohistochemistry against Ki67 protein was performed. Figure 3A shows the micrographs of Ki67 positive cells in the studied groups at three time points on days 7, 14 and 21. Quantitative evaluation of proliferating cells on day 7 showed that it was significantly higher in phenytoin, JRL 2%, and JRL 5% groups compared to control and eucerin groups (both, *P* < *0.05*, *P* < *0.05*, and *P* < *0.001*, respectively). On day 14, density of Ki67 positive cells was significantly more in JRL 2% and JRL 5% groups compared to control (*P* < *0.05* and *P* < *0.001*, respectively) and eucerin (*P* < *0.05* and *P* < *0.01*, respectively) groups. Finally, on day 21, the results indicated that the density of Ki67 positive cells in phenytoin, JRL 2%, and JRL 5% groups was significantly higher compared to control (*P* < *0.05*, *P* < *0.01*, and *P* < *0.001*, respectively) and eucerin (*P* < *0.05*, *P* < *0.05* and *P* < *0.001*, respectively) groups. (Fig. 3B).

### qRT-PCR gene expression

**Fig. 4:**
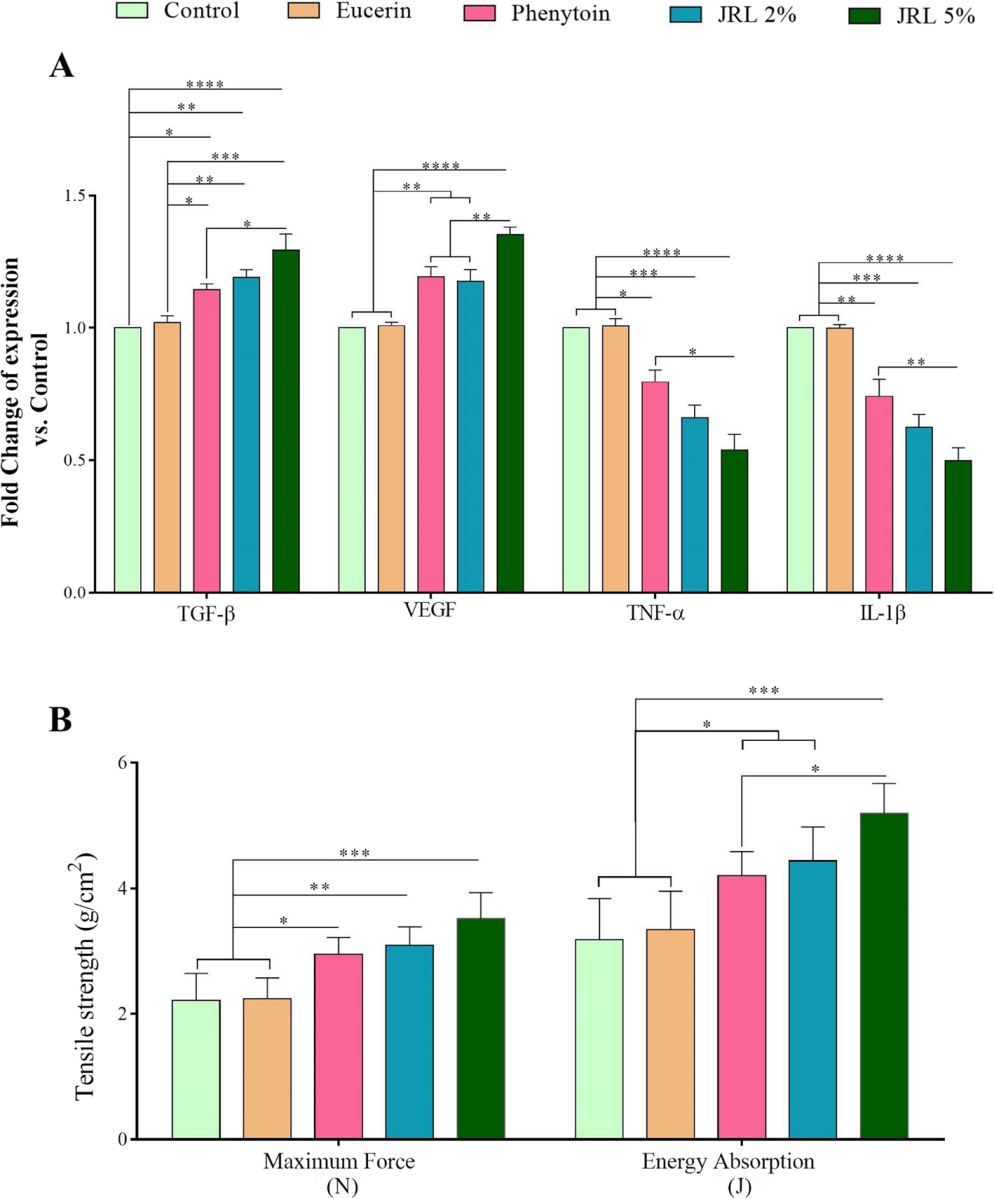
Effects of topical administration of *Juglans regia* L. leaf extract on gene expression and biomechanical characterizes. **A** The expression level of genes effective in proliferation and regeneration (TGF-β), angiogenesis (VEGF) and inflammation (TNF-α and IL-β) on day 7. **B** Biomechanical characteristics assessed on day 21. Data are represented as Mean ± SD. *P < *0.05*; **P < *0.01*, ***P < *0.001*, ****P < *0.0001*

To assessment the effects of *Juglans regia* L. leaf extract on wound healing at molecular levels, we determinate the amount of transcripts for TGF-β and VEGF genes (involved in regeneration and angiogenesis, respectively) as well as TNF-α and IL-1β genes (involved in inflammation) on day 7 (Fig. 4A). The results showed that the expression of TGF-β gene in phenytoin, JRL 2%, and JRL 5% groups in comparison to control (*P* < *0.05*, *P* < *0.01*, and *P* < *0.0001*, respectively) and eucerin (*P* < *0.05*, *P* < *0.01*, and *P* < *0.001*, respectively) groups considerably was higher. Moreover, TGF-β gene in JRL 5% group was significantly upregulated compared to phenytoin group (*P* < *0.05*).

Evaluation of VEGF gene expression among the study groups showed that phenytoin, JRL 2%, and JRL 5% groups had higher expression levels compared to the control and eucerin groups (both, *P* < *0.01*, *P* < *0.01*, and *P* < *0.0001*, respectively). Also, the levels of VEGF gene expression in the JRL 5% group was significantly higher compared to the phenytoin and JRL 2% groups (both, *P* < *0.01*).

Furthermore, evaluation of inflammatory genes among the study groups showed that phenytoin, JRL 2%, and JRL 5% groups in comparison to control and eucerin groups (both, *P* < *0.01*, *P* < *0.01*, and *P* < *0.0001*, respectively), the expression of TNF-α gene was significantly lower.

Also, regarding the IL-1β gene, similar results were obtained such that the phenytoin, JRL 2%, and JRL 5% groups had lower expression levels compared to the control and eucerin groups (both, *P* < *0.01*, *P* < *0.001*, and *P* < *0.0001*, respectively). Comparing the results between the treatment groups also showed that the JRL 5% group had lower expression levels of TNF-α and IL-1β genes compared to the phenytoin group (*P* < *0.05* and *P* < *0.01*, respectively).

### Biomechanical characterizes

To evaluate the consistency of the healing tissue, the skin of the repaired wounds was evaluated by tensiometery on day 21. The results are shown in Fig. 4B. We found that phenytoin, JRL 2%, and JRL 5% groups had significantly higher maximum force (*P* < *0.05*, *P* < *0.01*, and *P* < *0.001*, respectively) and energy absorption (*P* < *0.05*, *P* < *0.05*, and *P* < *0.001*, respectively) compared to both control and eucerin groups. Also, the comparison of mechanical properties between the treated groups showed that JRL 5% group in comparison to phenytoin group, had significantly higher energy absorption (*P* < *0.05*).

## Discussion

In the present study, the effects of topical administration of *Juglans regia* L. leaf extract ointment in two concentrations of 2 and 5% in diabetic wound healing were investigated. In general, our results showed that the extract used in both concentrations had clear and significant effects on all wound healing parameters compared to the control and eucerin groups. However, these changes were more considerable in the 5% extract group. Also, in the present study, the phenytoin group as a reference drug was compared with other groups. Although phenytoin was better compared to the control and eucerin groups, it was less effective compared to the extract groups, especially the 5% extract.

In the present study, in order to accurately evaluate the interventions performed in diabetic wound healing, evaluations were performed in three time periods. The seventh day was due to the overlap between the inflammatory and proliferative phases, the fourteenth day was due to the overlap of the proliferative phases and the beginning of the regeneration phase, and the 21st day was due to the peak of tissue regeneration and maturation [10, 11, 13].

Prolonged inflammation is one of the most important challenges in the chronic wound healing process [35]. Early in the wound healing process, some inflammatory cytokines, such as TNF-α and IL-1β, are secreted by some local cells, such as macrophages and cause inflammatory cells such as neutrophils to be absorbed into the wound site and the normal inflammatory phase begins [36]. However, excessive secretion of these cytokines prolongs the inflammatory phase, resulting in chronic ulceration [37]. In this regard, in the present study, two methods were used to evaluate the anti-inflammatory properties of treated regimens including stereological (neutrophils density), and molecular (TNF-α and IL-1β gene expressions) assessments. The results showed that the density of neutrophils and expression levels of TNF-α and IL-1β genes in the phenytoin, JRL 2%, and JRL 5% groups were significantly reduced compared to control and eucerin groups. However, in all cases, this reduction was more pronounced in the JRL 5% group in comparison to control and eucerin groups. Moreover, the comparison of the inflammatory results between extract groups and phenytoin group indicated a decrease in inflammation in the extract groups and these changes were significant in the JRL 5% group in all cases.

In this regard, our laboratory previously reported that *Juglans regia* L. leaf extract has significant anti-inflammatory effects and can significantly reduce the levels of inflammatory proteins such as COX-2, PARP and iNOS and prevent the development of diabetes complications [26–28]. On the other hand, Gharaboghaz et al. documented that there is a cross-link between the inflammation and antioxidant statues in wound area [38]. In this regard, we previously reported that *Juglans regia* L. leaf extract has very high antioxidant properties due to the presence of rich phenolic and flavonoid compounds [29]. Therefore, based on the our previous reports about antioxidative capacity of *Juglans regia* L. leaf extract, it is only logical to conclude that the antioxidant status can significantly suppresses the excessive inflammatory reactions.

The presence of some cytokines such as TGF-β in the wound bed can play a key role in healing. This cytokine has been shown to stimulate proliferation of keratinocyte and fibroblasts and regenerate the ECM [10]. Our results clearly showed an increase in TGF-β gene expression in the phenytoin, JRL 2%, and JRL 5% groups in comparison to control and eucerin groups. Furthermore, the comparison of TGF-β gene expression between the extract and phenytoin groups indicated that the level of this gene was more upregulated in the extract groups, and this difference was significant in the JRL 5% group compared to the phenytoin group.

Furthermore, Desmouliere et al. reported that TGF-β induces the differentiation of fibroblast cells into myofibroblasts and accelerates wound closure [39]. Also, TGF-β was reported that promote the proliferation of local cells such as fibroblasts and keratinocytes [10, 40]. Evaluation of fibroblasts density, volumes of newly formed epidermis and dermis, and cell proliferation in new dermis in present study indicated that their levels in the phenytoin, JRL 2%, and JRL 5% groups were considerably increased compared to control and eucerin groups. However, these changes were more pronounced in the JRL 5% group. Also, the comparison between extract and phenytoin groups indicated that the volumes of newly formed epidermis and dermis and the density of proliferating cells were higher in the JRL 5% group compared to the other two groups, and in some cases this difference was statistically significant.

Among the most important causes of delay in diabetic wound healing are hypoxia and lack of proper blood supply to the wound site [41]. Studies have shown that these factors are due to reasons such as vasculopathy and oxidative stress occurred in local endothelial cells that inhibited angiogenesis [35, 42]. Among these, one of the most important factors that play a key role in angiogenesis is VEGF. This factor is produced by localized cells such as fibroblasts, however, during diabetes due to hyperglycemia followed by oxidative stress, the ability of these cells significantly reduced [43]. Therefore, our hypothesis was that the use of a combination with antioxidant properties and blood sugar reduction can greatly reduce the occurrence of local oxidative stress and then increase the expression of VEGF factor and angiogenesis in the site. Our results confirmed this hypothesis that the levels of VEGF gene expression as well as the density of blood vessels in new dermis were significantly higher in the treated groups, especially in JRL 5% group compared to control and eucerin groups.

Collagen is one of the main structures of the ECM and determines the consistency of tissue [44]. These fibers are produced by fibroblasts in the dermis, however, during diabetes their production and quality are significantly reduced [9]. However, our hypothesis was that the use of *Juglans regia* L. leaf extract, as a strong antioxidant, can increase collagen production by controlling oxidative stress in fibroblast cells. In addition, in order to confirm the increase in collagen density in new dermis, the wound closure rate at the studied time intervals as well as the consistency of the repaired tissue at the end of the study were evaluated. The results showed that the collagen density, wound closure rate and wound strength in treated groups, especially the JRL 5% group, were significantly higher than control and eucerin groups. Comparing the results between the extract and phenytoin groups indicated that the effects of the extract, especially JRL 5%, were better, and in some cases the difference between them was significant.

In present study, there are two main limitations. First, the sample size should be larger for more experimental integrity. The second limitation is the use of the rat model for studying human wound healing. As the histological structures are different in human and rats, the healing characteristics have some differences between them. Contraction plays an important role in wound healing in rats [45]. However, the wounds of human mainly heal by re-epithelialization. Although the panniculus carnosus which plays an important role in wound contraction in rats has been removed after debridement, the involvement of contraction did not completely exclude in the present study.

## Conclusion

The present study demonstrated that topical administration of *Juglans regia* L. leaf extract significantly accelerate wound healing in compared to control and eucerin groups, however, these changes were considerable in the JRL 5% group. Considering the beneficial effects of *Juglans regia* L. leaf extract in other complications of diabetes that have been reported previously and our findings in present study, it is suggested to conduct human studies to confirm its effectiveness.

## Supplementary Information

**Additional file 1: Supplementary Fig 1.** Stereological analyses for newly formed of epidermis and dermis volumes, numerical cells density and blood vessel density.

## Data Availability

All the data is available with Prof. Ali Reza Khalatbary, reasonable request will be responded with supplementary raw-data.

## Abbreviations

ECM: Extracellular matrix
TNF-α: Tumor necrosis factor-alpha
IL-1β: Interleukin-1beta
TGF-β: Transforming growth factor-beta
VEGF: Vascular endothelial growth factor
STZ: Streptozotocin
IP: Intraperitoneal
FBS: Fast blood sugar
MT: Mallory's trichrome
DAB: Diaminobenzidine tetrahydrochloride
PBS: Phosphate buffered saline
qRT-PCR: Quantitative real-time polymerase chain reaction
COX-2: Cyclooxygenase-2
iNOS: Inducible nitric oxide synthase
PARP: Poly (ADP-ribose) polymerase
