# Supplementary material for: Topical administration of Juglans regia L. leaf extract accelerates diabetic wound healing
Source: BMC Complement Med Ther. 2022 Oct 3;22:255. doi: 10.1186/s12906-022-03735-6 (PMC9528103; doi:10.1186/s12906-022-03735-6)
Supplement: Supplementary file 1 — Additional file 1: Supplementary Fig 1. Stereological analyses for newly formed of epidermis and dermis volumes, numerical cells density and blood vessel density. [file 12906_2022_3735_MOESM1_ESM.pdf]

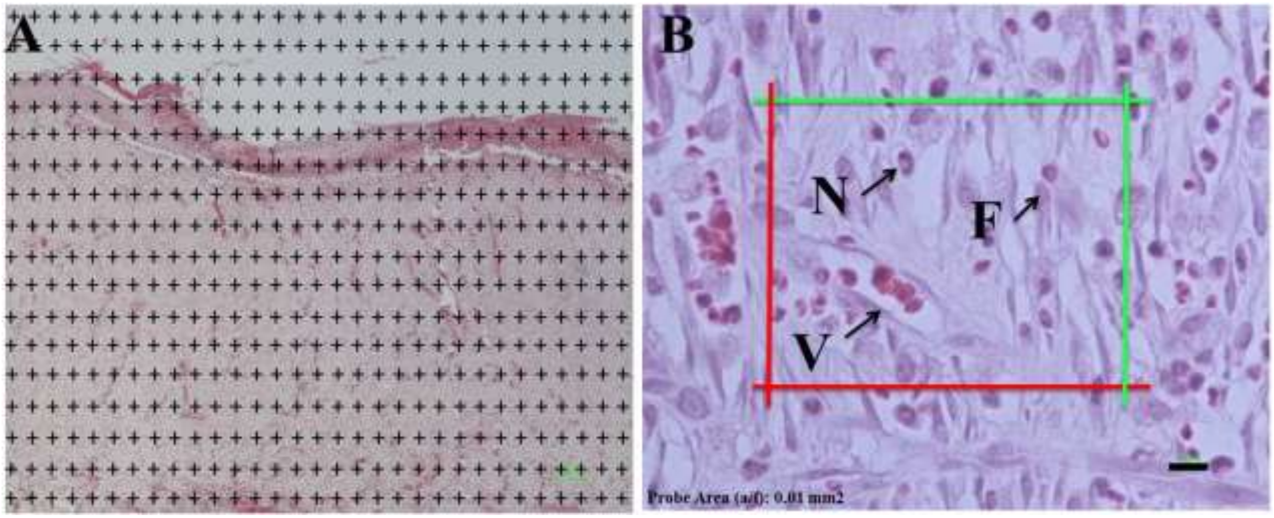


**Supplementary Fig 1.** Stereological analyses for newly formed of epidermis and dermis volumes, numerical cells density and blood vessel density.

Estimation the volume of the new epidermis and dermis using the Cavalieri method (A). For this purpose, wound area tissue is sectioned exhaustively and cut in parallel sections with a fixed distance. Next, A point grid is superimposed on each section (like above photo), and all points falling within the new epidermis and dermis are counted and the area associated with each point is multiplied with the total number of points and the distance between sections according to following equitation:

$$V_{total} = \sum P \times \frac{a}{p} \times \mathrm{t}$$

The ΣP, the sum of the points counted, the a/p ($mm^2$) is defined as the area related with each point projected on skin tissue, and t (mm) is recognized as the distance between the selected sections perceivably.

Estimation the numerical cells and blood vessel density (B). wound area tissue is sectioned exhaustively and cut in parallel sections with a fixed distance. Next, the sections are placed under a microscope at ×100 magnification. In each section, 5 areas, 4 of which were in the upper and lower right and left corners, respectively, as well as the center of the tissue, by a standard stereology probe with dimensions of 0.01 $mm^2$ attached to the microscope monitor were counted. According to the standard method, the cells and vessels located on the upper and right lines (green line) of the probe were counted, and the cells and vessels located on the left and lower lines (red line) were not counted. Therefore, for each section, a total of 5 numbers were calculated for each cell and blood vessel, and their sum was considered as the total number for that section. Finally, numerical density (Nv) of the fibroblasts and neutrophils in the healing bed, using following equitation:

$$N_v = \frac{\sum Q}{\sum P \times h \times \frac{a}{f}} \times \frac{t}{BA}$$

The ΣQ: the number of nuclei, h (µm): height of the dissector, Σp: the total number of the counted frames, a/f ($mm^2$): frame area, BA (µm): block advance of the microtome (set at 20 µm), t (µm): real sectional thickness.
Also, for measuring length density of the blood vessels, following equitation was used:

$$L_v = \frac{2\sum Q}{\sum P \times \frac{a}{f}}$$

The ΣP: total number of the counted frames; ΣQ: total number of the vessel profiles counted per skin; a/f ($mm^2$): counting frame area.

**F**; fibroblast **N**; neutrophil **V**; blood vessel
